# The Effect of Heat Treatment on a 3D-Printed PLA Polymer’s Mechanical Properties

**DOI:** 10.3390/polym15061587

**Published:** 2023-03-22

**Authors:** Mariam Shbanah, Márton Jordanov, Zoltán Nyikes, László Tóth, Tünde Anna Kovács

**Affiliations:** 1 Doctoral School on Materials Sciences and Technologies, Óbuda University, Bécsi út 96/B, 1034 Budapest, Hungary; 2 Bánki Donát Faculty of Mechanical and Safety Engineering, Óbuda University, Népszínház u. 8., 1081 Budapest, Hungary; 3 Department of Informatics, Milton Friedman University, Kelta u. 2., 1039 Budapest, Hungary

**Keywords:** additive process, PLA, heat treatment, tensile test, microscopy

## Abstract

Three-dimensional printing is a useful and common process in additive manufacturing nowadays. The advantage of additive polymer technology is its rapidity and design freedom. Polymer materials’ mechanical properties depend on the process parameters and the chemical composition of the polymer used. Mechanical properties are very important in product applicability. The mechanical properties of polymers can be enhanced by heat treatment. Additive-manufactured PLA’s mechanical properties and structure can be modified via heat treatment after the 3D printing process. The goal of this research was to test the effect of heat treatment on the mechanical and structural parameters of additive-manufactured PLA. This was achieved via the FDM processing of standard PLA tensile test specimens with longitudinal and vertical printing orientations. After printing, the test specimens were heat-treated at 55 °C, 65 °C and 80 °C for 5 h and after being held at 20 °C for 15 h. The printed and heat-treated specimens were tested using tensile tests and microscopy. Based on the test results, we can conclude that the optimal heat treatment process temperature was 65 °C for 5 h. Under the heat treatment, the test specimens did not show any deformation, the tensile strength increased by 35% and the porosity of the PLA structure decreased.

## 1. Introduction

Additive manufacturing is an advanced manufacturing process that can be used to rapidly prepare prototype components, innovate a new geometry or replace parts. PLA is a very useful and common polymer material that is widely used for the additive manufacturing process. PLA is a biodegradable and biocompatible thermoplastic polymer [1,2]. Melt polycondensation in PLA processing can be carried out without organic materials or a solvent. This method is simple, making the technology cheap, but the sensitivity of the reaction conditions is a major problem [3,4]. PLA is considered a bioplastic because it is produced from materials derived from renewable biomass products. The thermal, mechanical and biodegradation characteristics of lactic acid polymers are known [5,6]; they are made up of lactic acid units, which are small organic acids similar to those found in many of the foods we encounter every day—think sourdough bread, yoghurt, soy sauce and, of course, corn. Anything with glucose in it can theoretically be converted into lactic acid molecules. Among other things, their low glass transition temperature makes PLA parts easy to melt and manipulate, and therefore easy to 3D print [7]. However, this low glass transition value is also the reason why PLA parts are relatively less resistant to ambient temperatures.

Additive manufacturing, also known as 3D printing, has been in use in the industry for around 30 years and in recent years has been used for rapid prototyping and small-batch production of plastic and metal products. The additive manufacturing technologies (FDM, EBF, DMLS, EBM, SHS, SLS, PP, LOM, SLA, DLP) use different but essentially similar processes to produce a three-dimensional shape. FDM is a complex additive manufacturing process with a large number of technical parameters that influence product quality and material properties, and the combination of these parameters is often difficult to understand [7,8]. The printing parameters, such as printing orientation, layer thickness, raster angle, raster width, air gap, infill density and pattern and feed rate, have a substantial effect on the quality of FDM-printed parts [9,10,11,12].

The machine builds the spatial object layer by layer. The starting point is always a virtual model, which the target software converts into interpretable instructions before printing [8]. These technologies are mainly used in rapid prototyping, where production times can be reduced from weeks to 1–2 days. Rapid prototyping can quickly complete the development phase, allowing the tool to be made for mass production or even 3D printed. Increasingly, 3D printing is being used for small batches or one-off items for the final product. FDM printers process plastic fibers wound on a spool by melting them and then printing them layer by layer on a printing platform [7,8,9,10,11,12]. Thanks to the compatibility of materials and their user-friendliness, FDM printers are among the most popular 3D printers on the market. Among the process parameters that are most relevant to the mechanical properties is the printing direction [7,13]. The mechanical parameters of additive-manufactured polymers can be enhanced with heat treatment after the printing process [14,15,16,17,18,19,20,21,22,23,24,25]. The material properties of 3D-printed samples can be characterized via material testing [26,27]. Several researchers have conducted tensile tests to determine FDM-printed and heat-treated specimens’ mechanical properties, measuring the increase in UTS (32%) by annealing the specimens at 90 °C for 1 h [25].

Polymer additive technology is nowadays widely used in many areas, such as the aerospace industry, automotive industry, food industry and healthcare industry [28]. PLA is a biodegradable polymer that is useful for several applications, and this material can be composted.

This study aimed to find a cheap and suitable process and material for the rapid fabrication of laboratory devices. The dimensions of the device were determined considering the geometry of the tested specimen. The choice of material was primarily based on the need to choose a material with low density and adequate strength, due to the manual handling required for this study. The aim was to identify the specifications of a complex technology that give the best result using the chosen material quality and production technology.

## 2. Materials and Methods

### 2.1. 3D Printing of the Test Specimens

**Figure 1:**
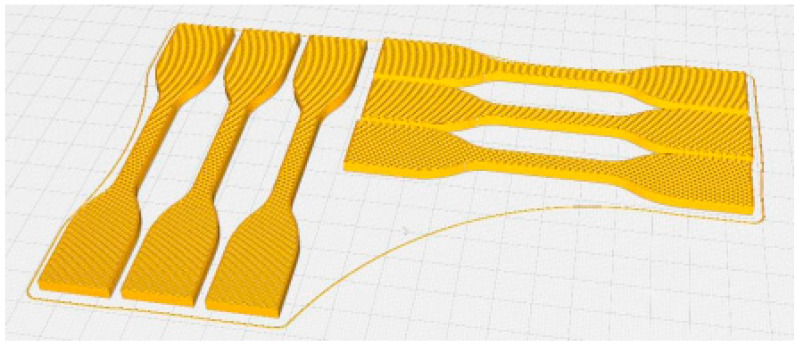
Test specimens on the work table.

The test specimens were prepared via the FDM process, and the printer used was the Ultimaker S5 Pro Bundle (Ultimaker, Utrecht, The Netherlands) with the Ultimaker Cura 5.0 software (Ultimaker, Utrecht, The Netherlands). The equipment used operated with efficient air filtration and humidity control. The filament material used was an Ultimaker PLA (RAL 1003) (Ultimaker, Utrecht, The Netherlands) with a 2.85 ± 0.10 mm diameter and 1.24 g/cm^3^ density on the base of the technical datasheet of the filament. The used printer’s maximal power was 600 W, and the position precision in XYZ axes was 6.9 μm, 6.9 μm and 2.5 μm. The layer resolution supported by Makerbot (Ultimaker, Utrecht, The Netherlands) was 20–600 μm. The used PLA filament’s ultimate tensile strength (UTS) was 49.5 MPa. The geometry of the printed specimens was suited to the ASTM D638 IV standard tensile test specimen (length 115 mm, width 6 mm and thickness 3.6 mm). The test samples were printed in vertical and longitudinal orientations. The test samples’ orientations on the printer table are shown in Figure 1.

**Figure 2:**
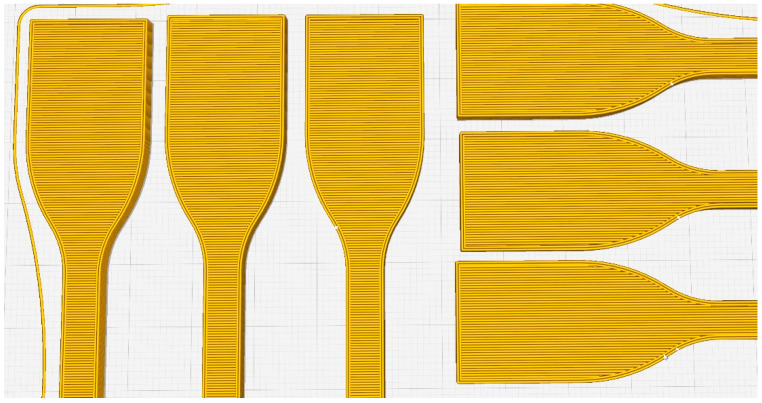
The orientation of the test specimens (vertical and horizontal).

The test samples were printed without support. The work table was preheated to 60 °C and the extruder melted the PLA filament at 200 °C. The printing speed was 35 mm/min and the prepared layer thickness was 0.2 mm. Printing parameters were chosen according to the filament and printer manufacturer’s recommendations. All specimens were made with 22 layers with constant orientation. The printing time for 3 vertically and 3 horizontally orientated specimens was 4.5 h. Vertical orientation refers to a position perpendicular to the test specimen’s length and the horizontal orientation corresponds to the test specimen’s length (Figure 2).

The cooling time of the test specimens after printing was 15 h. The cooling process was carried out in open air at room temperature (20 °C) in standard humidity conditions.

### 2.2. Heat Treatment Process

**Figure 3:**
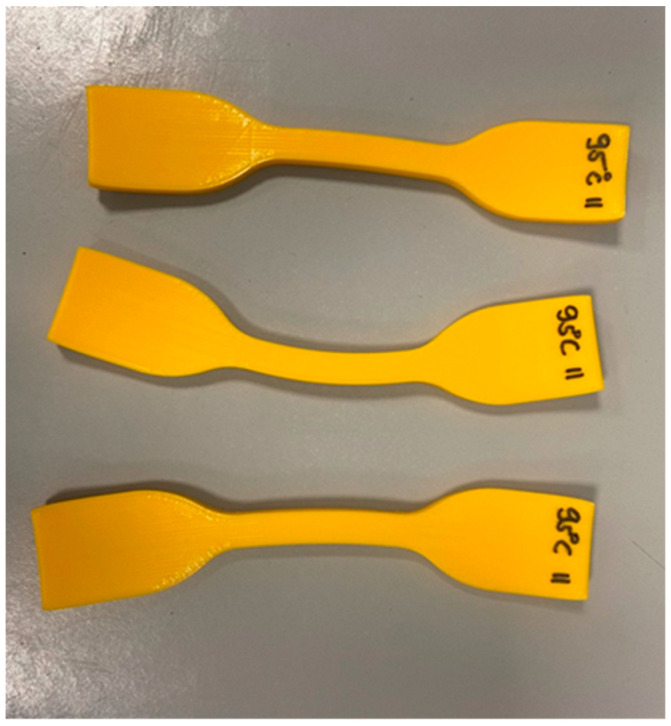
Deformed specimens after heat treatment.

The heat treatment was carried out for 15 h and took place in a precision furnace preheated to a standard temperature. The heat treatment temperatures were 55 °C, 65 °C, 80 °C and 95 °C. Cooling was carried out in the open air at room temperature for all specimens for 15 h. After the cooling period, all test specimens were tested via visual inspection and size control using a caliper. After the heat treatment process, the specimens heat-treated at 95 °C were significantly deformed. The heat-treated test specimens’ deformation can see in Figure 3.

The other heat-treated specimens did not show any deformation and kept their original geometry and sizes. The deformed specimens were not examined further.

### 2.3. Tensile Test

**Figure 4:**
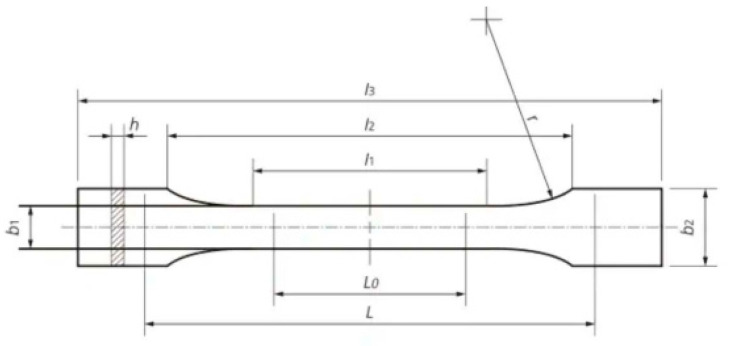
ASTM D638 IV standard test specimen design.

The tensile test was conducted using a mechanical testing machine. We measured the maximal force ($F_{\max}$) and determined the tensile strength using Equation (1).

(1) $$R_{m}=\frac{F_{\max}}{S_{0}}\;[\mathrm{MPa}]$$

where $S_{0}$ is determined using Equation (2):

(2) $$S_{0}=b_{1}\cdot h\;[{\mathrm{mm}}^{2}]$$

where b_1_ is the width and h is the thickness of the test specimen. The design of the standard test specimen is shown in Figure 4.

A tensile test was carried out on the printed test specimens to determine their tensile strength as a function of the orientation. Five specimens in both orientations were tested. The results (vertical orientation, V, horizontal orientation, H) of the tests are shown in Table 1. The tensile test was carried out at room temperature in climatized environments.

**Figure 5:**
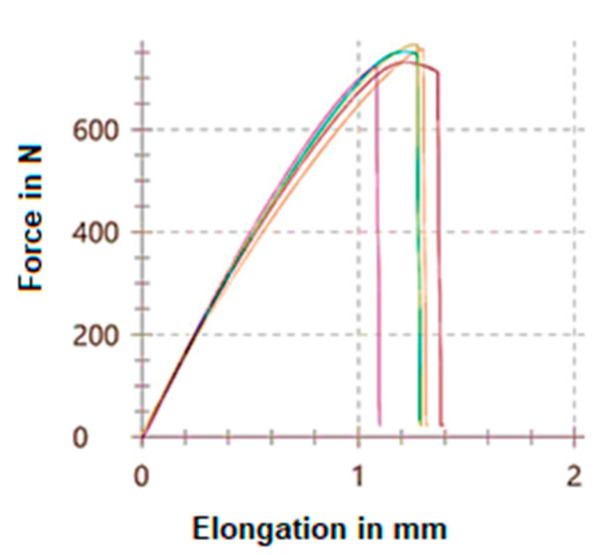
Tensile curves of the vertically orientated test specimens.

**Figure 6:**
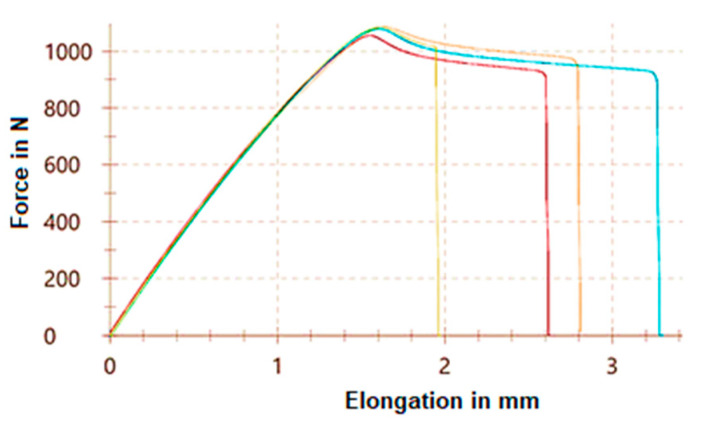
Tensile curves of the horizontally orientated test specimens.

The tensile curves determined in the case of the vertically orientated test specimens are shown in Figure 5 and the horizontally orientated test specimens in Figure 6.

It can be concluded that the tensile test results depended on the test specimens’ printing orientation. In the case of the horizontal orientation, the average tensile strength was 51.25 MPa, and in the case of the vertical printing orientation, it was 35.6 MPa. These tests were conducted to determine the printed specimens’ tensile strength as a function of the printing orientation.

### 2.4. Microscopy

**Figure 7:**
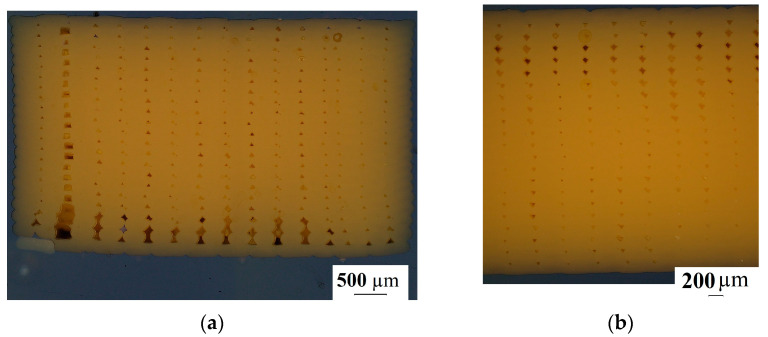
The test specimens’ cross-sections. (**a**) Perpendicular cross-section of a horizontally printed specimen; (**b**) perpendicular cross-section of a vertically printed specimen.

The printing efficiency was tested using an Olympus DSX 1000 (Olympus Corporation, Tokyo, Japan) microscope. The cross-sections of the vertically and horizontally orientated and heat-treated test specimens were tested. The samples were prepared for microscope observation via metallographic preparation, grounding and polishing. The cross-sections of the horizontally and vertically orientated test specimens are shown in Figure 7. The crossing direction was perpendicular to the printing direction of the test specimens.

### 2.5. Shore D Test

The hardness of the specimens was determined using a Shore D tester. The test was conducted at room temperature according to ISO 868. The technical datasheet of the filament gives 83 (D Shore) as a characteristic value of the 3D-printed state, as determined using a durometer (Innovatest SHD0002, Innovatest Europe BV, Maastricht, The Netherlands). There was no reference value for the direction of printing.

## 3. Results and Discussions

The goal of the experiments was to determine the heat-treated specimens’ tensile strength and structure as a function of the heat treatment in the case of both printing orientations. The heat treatment was carried out as described above at 55 °C, 65 °C, 80 °C and 95 °C. The latter temperature, 95 °C, led to deformation (see Figure 3) of the specimens; these were not examined further.

### 3.1. Heat-Treated Specimens’ Tensile Test Results

The other specimens’ tensile test results are summarized in Table 2. The labels in the table below are explained as follows: the first digit shows the printing orientation, where V is vertical and H is horizontal; the second digit, h, indicates that these specimens were heat-treated; and the third digit is the temperature of the heat treatment in °C.

Table 2 summarizes the tensile test results of the printed and heat-treated specimens. Based on the UTS results in the case of the PLA and printing process parameters used, the heat treatment at 65 °C led to the best performance for the vertically printed specimens and the heat treatment at 80 °C led to the best results for the horizontally printed specimens. Between the 65 °C and 80 °C heat treatments in the case of the horizontally printed specimens, there was a 0.5 MPa difference in UTS.

Numerous research reports suggest that the reason PLA’s mechanical properties are enhanced as a function of heat treatment is the improvement in the crystallinity of PLA. This refers to heat treatments conducted between 90 °C and 100 °C [29,30].

### 3.2. Tensile Test Resulted in Ruptured Surfaces

**Figure 8:**
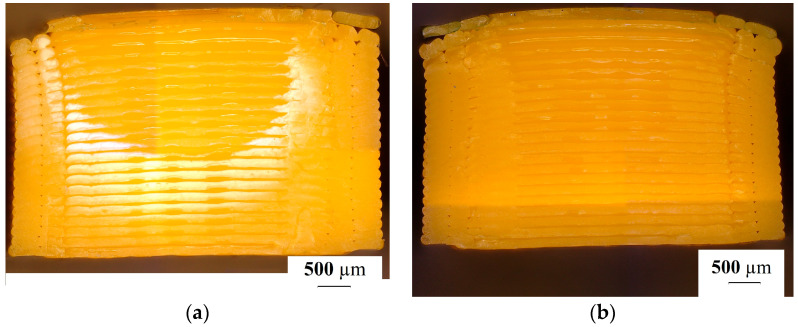
The ruptured surface of the vertically printed test specimens after the tensile test. (**a**) A specimen that did not undergo heat treatment; (**b**) specimen heat-treated at 65 °C.

After the tensile test, the specimens’ surfaces were examined via visual inspection and microscopy. The microscopy test showed differences as a function of the printing direction and the heat treatment in the extent of damage to specimens’ surface. Only the printed specimens and those heat-treated at 65 °C were tested because, according to the tensile test results, the vertically printed specimens showed the highest UTS. Figure 8 shows the vertically printed specimens’ ruptured surface.

**Figure 9:**
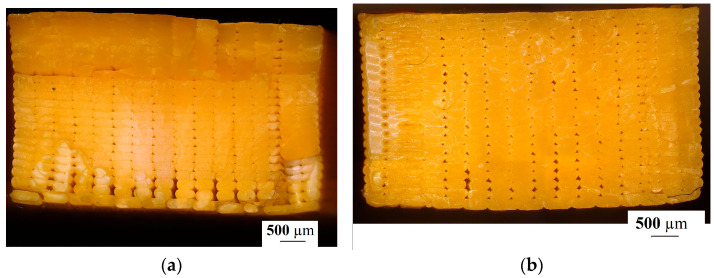
The ruptured surface of the horizontally printed test specimen after the tensile test. (**a**) A specimen that did not undergo heat treatment; (**b**) a specimen heat-treated at 65 °C.

The ruptured surfaces of the horizontally printed specimens are shown in Figure 9, where Figure 9a shows the non-heat-treated specimen and Figure 9b shows the heat-treated specimen.

The ruptured surfaces show the heat treatment effects. The surfaces had some brittle fractures (Figure 9c).

**Figure 10:**
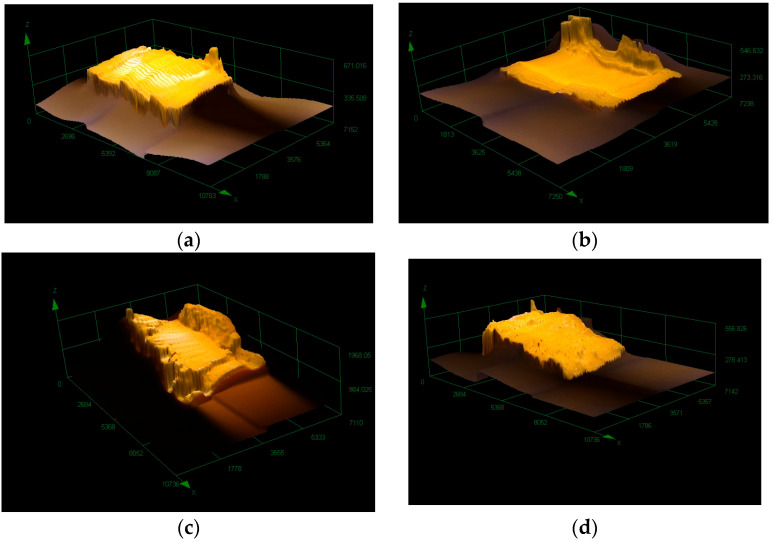
The ruptured surface of the horizontally and vertically printed test specimens after the tensile test. (**a**) The ruptured surface of a vertically printed specimen; (**b**) the ruptured surface of a vertically printed and heat-treated specimen; (**c**) the ruptured surface of a horizontally printed specimen; (**d**) the ruptured surface of a horizontally printed and heat-treated specimen.

The 3D pictures of the ruptured surfaces better show the difference between the heat-treated and non-heat-treated specimens (Figure 10). The heat treatment temperature was 65 °C.

### 3.3. Microscopy of the Specimen Cross-Section

**Figure 11:**
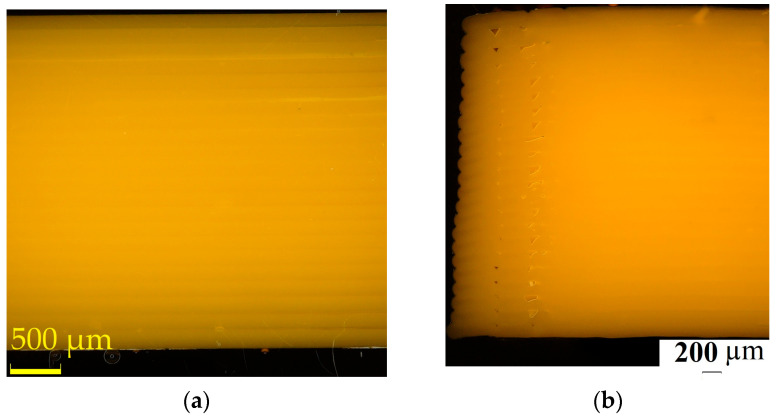

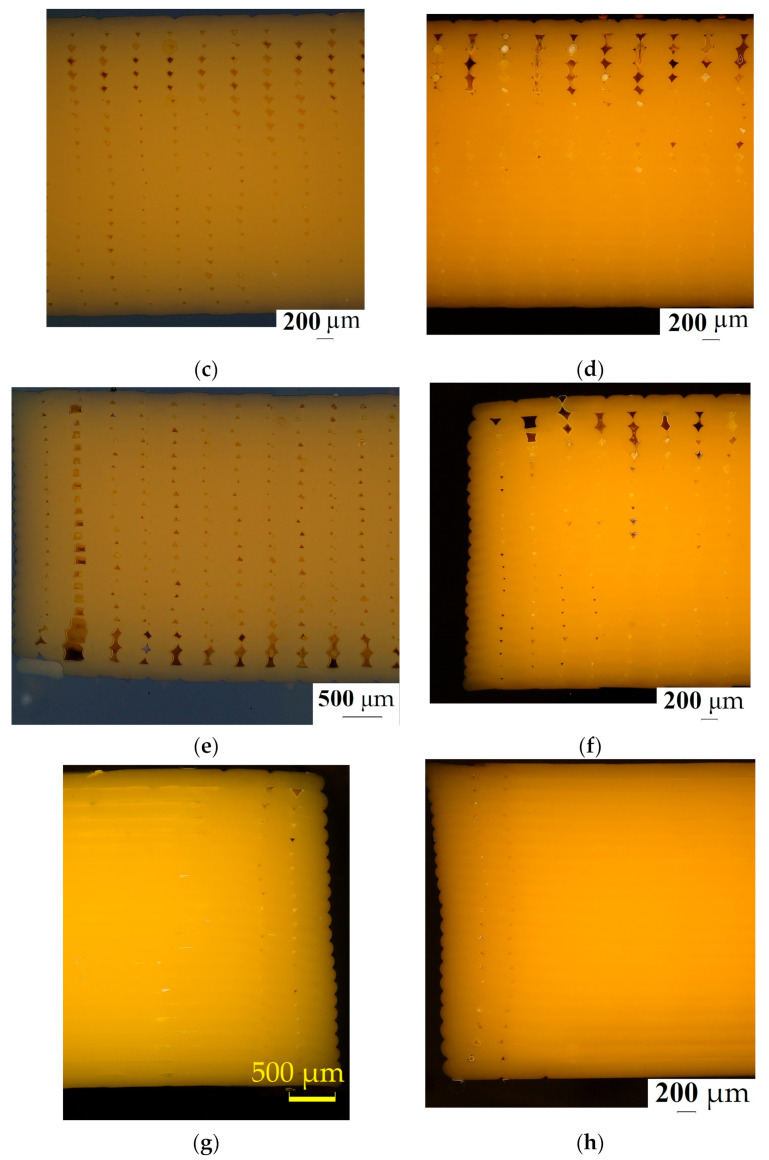
The microscopy tests of the printed and heat-treated specimens. (**a**) The vertically printed specimen cut parallel to the printing orientation; (**b**) the vertically printed specimen cut parallel to the printing orientation, after heat treatment at 65 °C; (**c**) the vertically printed specimen cut perpendicular to the printing orientation. (**d**) The vertically printed specimen cut perpendicular to the printing orientation, after heat treatment at 65 °C; (**e**) the horizontally printed specimen cut perpendicular to the printing orientation; (**f**) the horizontally printed specimen cut perpendicular to the printing orientation, after heat treatment at 65 °C; (**g**) the horizontally printed specimen cut parallel to the printing orientation; (**h**) the horizontally printed specimen cut parallel to the printing orientation, after heat treatment at 65 °C.

The microscopy results of the printed and the heat-treated test specimens’ cross-section are shown in Figure 11. Samples from both horizontally printed and vertically printed specimens were cut in two directions for microscopic examination. The printed and heat-treated specimens’ cross-sections were tested.

Figure 11 shows the structure of the printed and heat-treated specimens as a function of the printing orientation and the cross-section cut direction. It can be seen that the heat treatment decreased the porosity of the samples. In the case of the printed samples’ cross-sections, we can see that the porosity was different between the top side and the back side of the specimens. During the printing process, the temperature was different in each layer. To achieve suitable mechanical properties and the lowest porosity possible in a printed structure, polymerization needs to be maintained, which requires suitable temperature and environmental properties.

### 3.4. Shore D Hardness Tests

The Shore D test results are collected in Table 3. The test was carried out on the parallel and perpendicular cross-sections of the seven horizontally and vertically printed specimens heat-treated at 65 °C.

The average hardness in the case of all specimens was in harmony with the datasheet data and with the literature [31]. The heat treatment at 65 °C led to a decrease in hardness compared to the hardness value after printing. The applied heat treatment resulted in softening of the PLA.

## 4. Conclusions

Mechanical properties’ dependence on the printing direction is well understood. In the introduced experimental study, the results confirm this fact. The tensile test results are in harmony with the literature data; the printed tensile specimens showed a variable UTS as a function of the printing orientation [31]. This work is devoted to developing a heat treatment process able to enhance the mechanical properties of 3D-printed PLA products. The heat treatment process modified the UTS in the case of both vertical and horizontal printing directions. The heat treatment at 65 °C led to the greatest increase in the UTS of the vertically printed specimens. In the case of the horizontally printed specimens, all heat treatments increased the UTS (Table 2).

Based on the visual and microscopy tests of surface rupture after the tensile test, we can see fractures in the surface (Figure 9 and Figure 10).

The microscopy tests of the printed specimens heat-treated at 65 °C showed relevant differences in the structure of the specimens. Figure 11 shows the printed and heat-treated specimens, where it can be seen that after the heat treatment, the porosity decreased in all test specimen structures. The size of the test specimens was controlled by using a caliper, so there was not a measurable difference before and after the heat treatment in the case of the treatments at 55 °C, 65 °C and 80 °C. We can conclude that the heat treatment at 95 °C caused deformation (Figure 3). The increase in the UTS could be explained by the improvement in the crystallinity as the literature suggests, but on the basis of the experimental results, this cannot be fully concluded, because the crystalline fraction was not determined in this study. The effectiveness of the heat treatment, which is in harmony with the literature, is likely to be due to the reduction in stress as a result of the heat treatment [25,32]. All test specimens contained 22 layers, each of which stayed at a different temperature during the printing process for different durations of time.

It can be declared that heat treatment is a suitable method for increasing the mechanical properties of PLA after the 3D printing process. The printing orientation of PLA is the most relevant factor influencing the UTS, but this can be increased by a suitable heat treatment.

## Figures and Tables

**Table 1:** The tensile test results of the vertically orientated, V, and the horizontally orientated, H, specimens.

Id. n.	b_1_ (mm)	h (mm)	S_0_ (mm^2^)	Fmax (N)	ΔL (mm)	Rm (MPa)
V	6.0	3.5	21.0	745.4	1.28	35.6
H	6.0	3.5	21.0	1075	2.65	51.25

**Table 2:** The tensile test results of the heat-treated vertically orientated (V) and horizontally orientated (H) specimens as a function of the heat treatment temperature.

Sample	$b_{1}\;(\mathrm{mm})$	h (mm)	$S_{0}\;({\mathrm{mm}}^{2})$	$F_{\max}\;(N)$	ΔL (mm)	Rm (MPa)
V	6.0	3.50	21.00	745.40	1.28	35.60
H	6.0	3.50	21.00	1075.00	2.65	51.25
Vh55	5.99	3.50	20.97	873.00	1.23	41.33
Hh55	6.02	3.50	21.05	1273.33	1.90	60.33
Vh65	6.00	3.51	21.18	969.33	1.47	46.00
Hh65	6.00	3.50	20.98	1403.33	2.00	67.00
Vh80	6.01	3.51	21.10	662.00	0.85	31.00
Hh80	6.01	3.50	21.03	1415.00	2.00	67.50

**Table 3:** The Shore D test results of the printed and the heat-treated (h65) vertically printed (V) and horizontally printed (H) specimens’ parallel (pa) and perpendicular (pp) cross-sections.

Sample	Hpa	Hpp	Vpa	Vpp	Hh65pa	Hh65pp	Vhpa	Vhpp
Shore D	83	82	82	83	80	81	81	80

## Data Availability

The data used for the research are available upon request.
